# Artificial Intelligence in Patient Education: ChatGPT for Penile Prosthesis Surgery Counseling, a Likert Scale Analysis

**DOI:** 10.1111/andr.70236

**Published:** 2026-04-23

**Authors:** Gabriele Tulone, Nicola Pavan, Rosa Giaimo, Alchiede Franco Simonato, Alfonzo Salzano, Davide Cosentino, Francesco Claps, Alchiede Simonato

**Affiliations:** ^1^ Department of Precision Medicine in the Medical, Surgical and Critical Care Area (Me.Pre.C.C.) University of Palermo Palermo Italy; ^2^ Oncological Urology Veneto Institute of Oncology IRCCS Padua Italy

**Keywords:** artificial intelligence, erectile dysfunction, patient education, penile prosthesis, urology

## Abstract

**Introduction:**

Erectile dysfunction (ED) significantly impacts quality of life. For patients unresponsive to conservative treatments, penile prosthesis implantation represents an effective solution with high satisfaction rates. However, the complexity and sensitive nature of the procedure often generate multiple patient concerns. Artificial intelligence (AI) chatbots such as ChatGPT are increasingly used for medical information, although concerns remain regarding accuracy and misinformation. This study aimed to evaluate the clarity, accuracy, and clinical appropriateness of ChatGPT's responses to frequently asked questions (FAQs) on penile prosthesis surgery.

**Methods:**

Fifteen representative FAQs were identified from patient forums and educational platforms. Questions were submitted to ChatGPT (GPT‐4, November 2024) in a single session. A panel of seven expert urologists evaluated responses using a four‐point Likert scale across four domains: clarity, medical accuracy, relevance, and risk of misinformation.

**Results:**

All responses achieved mean scores above 3, indicating overall clinical acceptability. The highest ratings were observed for relevance (3.40 ± 0.54) and clarity (3.37 ± 0.57). Slightly lower scores were reported for medical accuracy (3.19 ± 0.67) and risk of misinformation (3.18 ± 0.59). A strong positive correlation was found between clarity and relevance (*ρ* = 0.80), while medical accuracy correlated with lower misinformation risk (*ρ* = 0.77).

**Conclusions:**

ChatGPT provides generally clear, relevant, and clinically acceptable responses for patient education in penile prosthesis surgery, although minor clarifications are often required. It may serve as a supportive tool to complement traditional counseling but cannot replace physician–patient communication, which remains essential for individualized care and informed decision‐making.

## 1 Introduction

Erectile dysfunction (ED) is a highly prevalent condition that significantly affects quality of life. It affects up to 52% of men aged 40–70 years, with severe forms in about 10% of cases [1, 2, 3]. Causes include metabolic, cardiovascular, neurological, and surgical conditions, with radical prostatectomy being a major risk factor, leading to postoperative ED rates up to 85% [4, 5]. For patients unresponsive to conservative therapies, penile prosthesis implantation offers a definitive and effective treatment option, with consistently high satisfaction rates when performed by experienced surgeons. However, the intimate and complex nature of this procedure generates numerous pre‐ and postoperative concerns, often related to device type, durability, complications, recovery, and sexual outcomes [6, 7]. In recent years, artificial intelligence (AI)–based chatbots, such as ChatGPT, have become increasingly accessible sources of medical information. These tools provide rapid responses to health‐related questions but raise concerns regarding accuracy, risk of misinformation, and potential impact on physician–patient communication [8]. Given the sensitive and stigmatized context of penile prosthesis surgery, reliable patient education resources are particularly important.

Recent studies have already explored the role of ChatGPT in patient education for penile prosthesis surgery, including the works by Shayegh et al. [9] and Schmidt et al. [10], which provided initial evidence on the quality and safety of AI‐generated information in this specific clinical setting. The present study builds upon this emerging literature using a structured methodological approach based on systematic patient‐derived questions and a multi‐domain expert evaluation with inter‐rater reliability analysis. The aim of this study was to evaluate the clarity, accuracy, and clinical appropriateness of ChatGPT's responses to frequently asked patient questions (FAQs) on penile prosthesis implantation, using structured assessment by a panel of expert urologists.

## 2 Materials and Methods

ChatGPT, developed by OpenAI, is an advanced large language model (LLM) that leverages AI to generate human‐like responses based on user input [11]. We conducted a cross‐sectional observational study to assess the accuracy, clarity, and clinical appropriateness of ChatGPT's responses to FAQs on penile prosthesis implantation.

### 2.1 Identification of Frequently Asked Questions

A structured search was conducted to identify FAQs related to penile prosthesis implantation. Questions were retrieved exclusively from publicly accessible online sources commonly used by patients seeking information on ED and penile prosthesis surgery. Specifically, the following patient forums and educational platforms were systematically reviewed: Inspire (urology and sexual health discussion boards), MedHelp (urology section), Patient.info (erectile dysfunction and penile implant forums), HealthUnlocked, and Reddit (subreddits r/ErectileDysfunction and r/Urology). In addition, FAQ sections from dedicated patient education websites, including those hosted by academic urology departments and professional urological associations, were reviewed.

An initial pool of approximately 80 questions was collected. Selection was performed in two stages: Screening by two independent reviewers (urology fellows) to remove duplicates and exclude questions not directly related to penile prosthesis surgery (e.g., general ED treatment, insurance coverage) and consensus review by the research team (seven urologists with ≥ 5 years of experience in prosthetic surgery, five of these reviewers are authors of the present manuscript) to ensure clinical relevance and representation across the perioperative journey. Final inclusion criteria: Question explicitly related to penile prosthesis implantation (preoperative, intraoperative, postoperative aspects). High frequency of recurrence across sources. Clinical relevance to patient counseling. Exclusion criteria: Non‐clinical questions (e.g., costs, insurance). Duplicates or questions with identical meaning. Questions too vague or not directly answerable. Following this process, 15 representative FAQs were selected (Table 2)

### 2.2 Chatbot Interaction and Evaluation

All 15 questions were submitted to ChatGPT (OpenAI, GPT‐4, November 2024 update) within a single conversation session to simulate a realistic patient–chatbot interaction.

ChatGPT (OpenAI, GPT‐4 version, November 2024 update) was selected for this study because it represents one of the most widely used and publicly accessible LLMs for health‐related information seeking. GPT‐4 has been extensively evaluated in prior peer‐reviewed studies assessing AI‐generated medical and surgical patient education content, allowing direct comparison with existing literature. The use of a single, well‐established model enabled a standardized and reproducible evaluation framework, minimizing heterogeneity related to differences in model architecture, training data, and response generation strategies. Although other LLMs (e.g., Gemini, Claude) are increasingly integrated into web‐based search engines and may influence real‐world patient information‐seeking behavior, a comparative multi‐model analysis would require a distinct study design and was beyond the scope of the present investigation. Future studies should directly compare multiple LLMs to assess variability in accuracy, clarity, and risk of misinformation across platforms.

Responses were collected in their original form without editing. The full ChatGPT‐generated responses are reported in Appendix A. A panel of seven urologists, each with more than 5 years of experience in prosthetic surgery, independently rated the responses on a four‐point Likert scale (1 = strongly disagree; 4 = strongly agree) across four dimensions:

‐ Clarity: how understandable and well‐structured the content is

‐ Medical Accuracy: consistency with scientific evidence and current guidelines

‐ Relevance: alignment with the patient's original question

‐ Risk of Misinformation: potential to mislead, alarm, or confuse the patient (Table 1)

**TABLE 1:** ChatGPT response rating scale.

Score	Category	Description
1	Strongly disagree	Strongly disagree
2	Disagree	Requires moderate clarification
3	Agree	Requires minor clarification
4	Strongly agree	Satisfactory content

*Note*: For the “Risk of Misinformation” domain, higher Likert scores indicate a lower perceived risk of misleading or unsafe content (i.e., 4 = minimal or no risk of misinformation; 1 = high risk of misinformation).

For the “Risk of Misinformation” domain, the Likert scale was intentionally constructed so that higher scores indicated a lower perceived risk of misleading or unsafe content. Specifically, a score of 4 corresponded to minimal or no risk of misinformation, whereas a score of 1 indicated a high risk of misleading or potentially harmful information. This directional structure was adopted to ensure consistency across evaluation domains, whereby higher scores uniformly reflected higher quality responses.

### 2.3 Statistical Analysis

For each domain, mean and standard deviation (SD) scores were calculated. Responses with an average score ≥ 3 were considered clinically acceptable. Correlation analysis between evaluation dimensions was performed using Spearman's rank correlation coefficient (*ρ*) to explore inter‐domain relationships. All analyses were conducted using Python (v.3.11) with the libraries *pandas* and *matplotlib*.

Inter‐rater reliability among the expert reviewers was assessed using the intraclass correlation coefficient (ICC). A two‐way random‐effects model with absolute agreement was applied, which is the recommended approach for evaluating agreement among multiple raters assessing the same items [12, 13]. ICC is specifically designed to quantify inter‐rater agreement and is more appropriate than internal consistency measures, such as Cronbach's α, in expert‐based evaluations [14, 15, 16].

### 2.4 Ethics

As no identifiable patient data were collected and all sources were publicly available, this study did not require formal ethics committee approval.

## 3 Results

**FIGURE 1:**
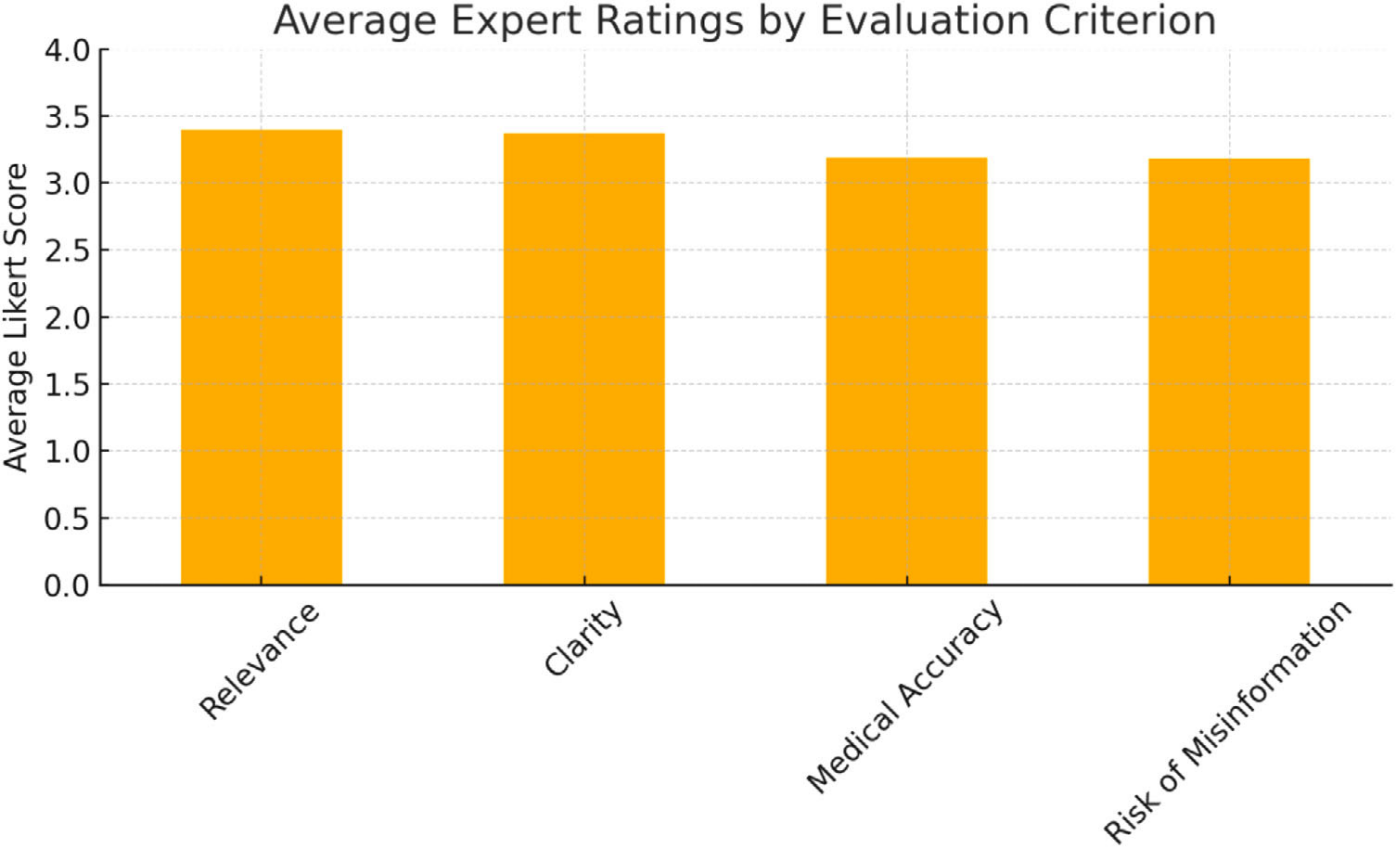
Average Likert scores per criterion.

**FIGURE 2:**
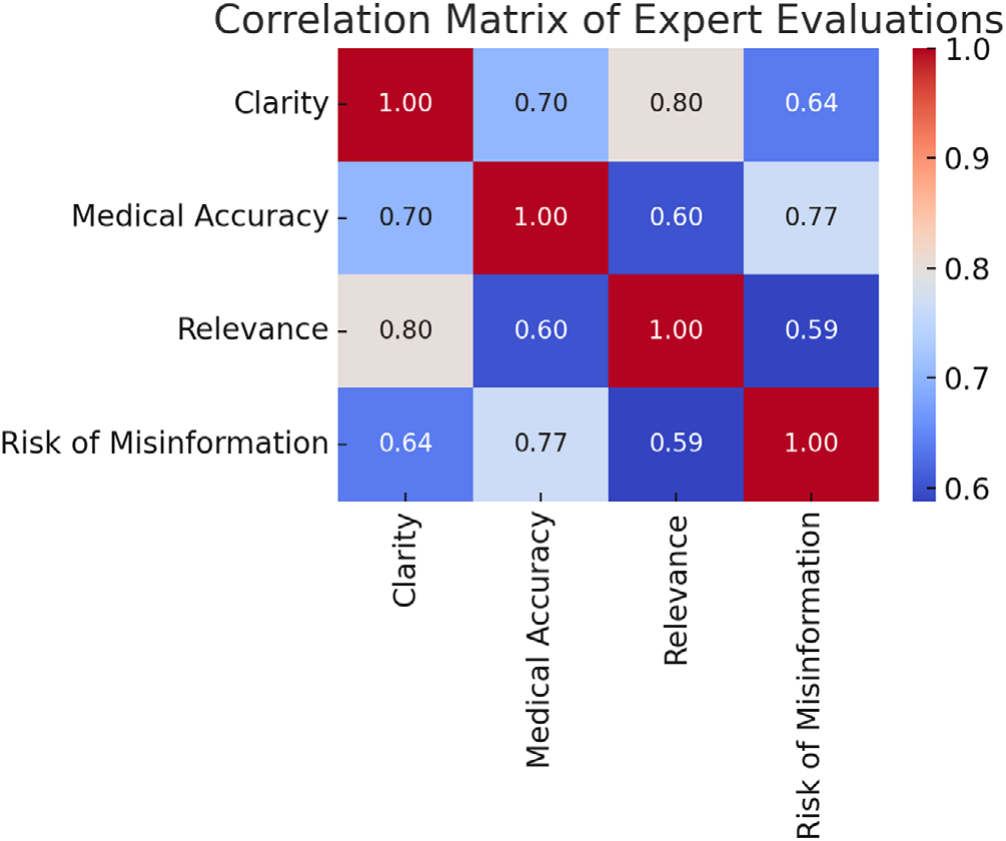
Correlation matrix of evaluations. Correlation matrix showing the relationships between evaluation domains. Higher scores in the “Risk of Misinformation” domain correspond to a lower perceived risk of misleading content; therefore, positive correlations indicate greater medical safety.

Fifteen patient FAQs regarding penile prosthesis implantation were submitted to ChatGPT within a single conversation session to simulate a realistic patient–chatbot interaction (Table 2). Responses were evaluated by a panel of seven urologists using a four‐point Likert scale. ChatGPT provided comprehensive answers to all questions (Table 3). Across the four evaluation domains, all responses achieved mean scores above 3, indicating overall clinical acceptability (Table 4). The highest ratings were observed for Relevance (mean = 3.40, standard deviation = 0.54), followed by Clarity (mean = 3.37, standard deviation = 0.57), while Medical accuracy (mean = 3.19, standard deviation = 0.67) and Risk of Misinformation (mean = 3.18, standard deviation = 0.59) scored slightly lower but remained positive. Correlation analysis demonstrated a strong positive association between Medical Accuracy and Risk of Misinformation (*ρ* = 0.77). This finding reflects the scoring structure of the evaluation framework, whereby higher scores in the “Risk of Misinformation” domain indicate a lower likelihood of misleading content. Accordingly, responses rated as more medically accurate were also perceived as safer and less likely to generate misinformation (Figures 1 and 2). As higher scores in the Risk of Misinformation domain indicate lower perceived risk, positive correlations reflect greater medical safety. The variability in response length suggests that the model attempts to adjust the depth and scope of information in relation to the question's perceived complexity. However, longer responses may also present challenges for patient comprehension, especially in settings with low health literacy. Inter‐rater reliability analysis demonstrated excellent agreement among the expert reviewers. The ICC showed a high level of consistency across ratings (ICC = 0.93; 95% CI, 0.82–0.98; *p* < 0.001), supporting the robustness and reproducibility of the expert‐based evaluation frammework (Table 5).

**TABLE 2:** FAQs asked by patients.

(1)	What is the best penile prosthesis and how to choose?
(2)	How does the three‐component penile prosthesis work?
(3)	What is the recovery time after penile prosthesis implantation?
(4)	What are the recommendations before and after penile prosthesis implantation surgery?
(5)	After how long can sexual intercourse be resumed after penile prosthesis implantation?
(6)	Is it necessary to use erection pills after penile prosthesis implantation?
(7)	Can you achieve orgasm with a penile prosthesis?
(8)	Can you resume physical activity after penile prosthesis implantation?
(9)	How long does a penile prosthesis last? Does a hydraulic penile prosthesis require replacement?
(10)	After penile prosthesis implantation surgery, will the penis be normal?
(11)	Can the sensitivity of the penis change after the prosthesis is placed?
(12)	What kind of erection will I be able to have after the placement of a penile prosthesis?
(13)	How long does sexual intercourse last with a penile prosthesis?
(14)	What are the potential complications of penile prosthesis surgery?
(15)	Is penile prosthesis placement surgery reversible?

**TABLE 3:** Word count.

Question	Topic	Word count
1	Type of prosthesis	241
2	How does a penile prosthesis work	88
3	Recovery time after surgery	214
4	Pre‐ and postoperative recommendation	143
5	Resume of sexual activity	132
6	Medication management	160
7	Reaching orgasm	253
8	Resuming physical activity after penile prosthesis implantation	170
9	Durability of the prosthesis	210
10	Potential cosmetic changes	239
11	Postoperative change in sensitivity	353
12	Kind of erection after implantation	478
13	How long does sexual intercourse last	279
14	Potential complication of the implantation	664
15	Reversibility of the surgery	335

**TABLE 4:** Descriptive statistics.

Dimension	Mean	Standard deviation
Relevance	3.40	0.54
Clarity	3.37	0.57
Medical Accuracy	3.19	0.67
Risk of Misinformation	3.18	0.59

**TABLE 5:** Inter‐rater reliability was assessed using the intraclass correlation coefficient (ICC).

Evaluation domain	ICC (A,15)	95% CI	Interpretation
Overall expert agreement	0.93	0.82–0.98	Excellent

A two‐way random‐effects model with absolute agreement and average measures was applied. ICC values > 0.90 indicate excellent agreement among raters.

## 4 Discussion

In recent years, the incorporation of AI into healthcare has accelerated considerably, particularly in areas such as diagnostics, personalized treatment, and patient engagement [18, 19, 20]. This study is the first structured evaluation of ChatGPT in the context of penile prosthesis counseling. Despite the high satisfaction rates associated with this surgery, many patients experience anxiety and uncertainty before the procedure, often leading to frequent preoperative questions concerning device types, durability, complications, recovery, and sexual outcomes [21, 22]. Because of the intimate nature of the surgery, some patients may be reluctant to raise these questions in person, making AI‐driven chatbots a potentially valuable complement to traditional counseling. Our analysis of 15 representative FAQs demonstrated that all four evaluation domains exceeded the clinical acceptability threshold, with the highest ratings for **Relevance** and **Clarity**. Slightly lower ratings for **Medical accuracy** and **Risk of misinformation** highlight the need for refinement. The strong positive correlation between clarity and relevance, and the inverse correlation between accuracy and misinformation risk, emphasize the interdependence of these domains in safe patient education. These findings are consistent with prior studies in thoracic [23, 24], orthopedic [25], and oncologic surgery [26], which reported that ChatGPT provides generally clear but sometimes inaccurate information. Similarly, our correlation results align with prior observations that clarity is closely linked to perceived utility [27], while inaccuracy increases the likelihood of misinformation [28]. Strengths of this study include the systematic selection of FAQs from real patient sources and structured evaluation by a multidisciplinary panel of experienced urologists. As shown by Shao et al. [29], ChatGPT's answers can vary depending on the phrasing of questions, contextual framing, or even the timing of the interaction However, variability in response length was observed, with some answers exceeding 600 words. While comprehensive content may reflect perceived complexity, excessive detail risks information overload and reduced retention, especially in patients with limited health literacy [7, 30]. Clinically, ChatGPT may serve as a useful adjunct to counseling by addressing common or repetitive questions and improving patient preparedness [31]. However, its role must remain complementary. Intimate and emotionally sensitive procedures such as penile prosthesis implantation require personalized physician–patient communication that AI cannot replace. Ethical oversight, transparency, and supervision remain essential to avoid misinterpretation and overreliance on AI tools [26].

Inter‐rater reliability was assessed using the ICC, which is considered the most appropriate statistic for evaluating agreement among multiple expert raters scoring the same items [12, 13]. Unlike measures of internal consistency such as Cronbach's α, ICC is specifically designed to assess agreement between raters and avoids methodological limitations when applied to expert‐based assessments [14, 15]. The excellent ICC observed in this study further supports the methodological robustness of the evaluation process.

Another relevant aspect concerns how misinformation is identified and quantified within expert evaluations. Although the Likert scale provides a standardized method for comparing domains, it may underestimate the clinical impact of subtle inaccuracies within AI‐generated responses. Even minor errors may disproportionately influence patient understanding, particularly in the context of intimate and technically complex procedures such as penile prosthesis implantation. Recent analyses have shown that LLMs can generate highly fluent but factually incorrect statements that patients may perceive as authoritative and therefore fail to recognize as inaccurate [17, 27].

Despite these limitations, the use of Likert‐based expert scoring remains methodologically appropriate at this stage. Several recent studies evaluating AI‐generated medical information—including applications in urology—have used structured expert ratings to assess clarity, accuracy, and informational safety [28, 29, 30, 31]. In addition, evidence from health communication research indicates that trained clinicians are capable of reliably detecting and judging even subtle forms of misinformation when using structured rating systems [32, 33, 34, 35].

Nevertheless, future studies should incorporate patient‐centered assessments to better quantify the real‐world impact of misinformation on comprehension, expectations, and decision‐making, as patients may be especially vulnerable to highly fluent but inaccurate AI‐generated content.

This study has several limitations. First, the analysis was conducted using responses generated within a single conversation session, without testing for temporal reproducibility or variation across different users or prompt styles. Second, evaluation was performed exclusively by clinical experts, without inclusion of patient perspectives such as perceived clarity, empathy, accessibility, or usefulness. Although expert review is essential for assessing medical accuracy, relevance, and safety, it does not fully capture how information is perceived and understood by patients. In particular, expert‐rated measures of clarity may not align with patient comprehension, especially among individuals with limited health literacy or heightened preoperative anxiety. Patients may interpret AI‐generated responses differently from clinicians, potentially overestimating their authority and failing to recognize subtle inaccuracies or ambiguities. Finally, some responses were excessively long, raising concerns about information overload, which may further hinder comprehension in vulnerable patient populations. For these reasons, expert‐based assessment should be regarded as a necessary but preliminary step, and future studies should incorporate patient‐centered outcome measures to better evaluate the real‐world effectiveness and safety of AI‐assisted patient education in penile prosthesis surgery.

## 5 Conclusion

ChatGPT provided responses rated as clear, relevant, and generally accurate, but with consistent need for minor clarifications. This study highlights both the promise and the current limitations of AI in sensitive areas of urology. Future research must include patient‐centered evaluations to determine its real impact on knowledge, anxiety, and decision‐making. Human oversight is critical to ensure medical accuracy, emotional appropriateness, and contextual sensitivity. Without personalized guidance, there remains a risk of misunderstanding or misinterpretation, particularly for patients with low health literacy or heightened emotional stress.

### 5.1 Statement of Contribution

What is already known:

Recent studies have evaluated the performance of LLMs in providing patient education content in urology, including penile prosthesis counseling. However, methodological heterogeneity and limited assessment of inter‐rater reliability remain challenges.

What this study adds:

This study provides a structured multi‐domain expert evaluation of ChatGPT‐generated responses in penile prosthesis counseling, incorporating formal inter‐rater reliability analysis using the ICC. Our findings contribute to the methodological framework for evaluating AI‐generated medical information in sensitive urological contexts.

## Author Contributions

**Alchiede Simonato and Nicola Pavan**: designed the study. **Gabriele Tulone**: performed the experiments, wrote the original draft and performed the statistical analysis. **Francesco Claps**: analyzed the data, and wrote the original draft. **Rosa Giaimo**: performed experiments. **Nicola Pavan and Alchiede Simonato**: reviewed and edited the manuscript; All the authors read and approved the final manuscript.

## Funding

The authors received no specific funding for this work.

## Ethics Statement

The authors have nothing to report.

## Conflicts of Interest

The authors declare no conflicts of interest.

## Appendix A

### A.1

**Response rating scale**

**Table:**

1. What is the best penile prosthesis and how to choose?	Choosing the best penile prosthesis depends on several factors, including your medical history, lifestyle, personal preferences, and budget. There are two main types of penile implants: 1. Inflatable penile prostheses (IPP) – Most popular and natural Three‐piece inflatable implant (most natural and functional) Pros: Provides a more natural erection and flaccid state, easier to conceal, and has the best rigidity. Cons: More complex surgery and higher risk of mechanical failure. Brands: AMS 700 (Boston Scientific), Titan (Coloplast) Two‐piece inflatable implant Pros: Easier surgery, fewer components. Cons: Less rigidity than three‐piece, not as natural. Brands: AMS Ambicor 2. Malleable (semi‐rigid) penile prostheses – Simpler and cheaper Pros: Simple, lower cost, easier surgery, good for patients with poor hand dexterity (e.g., after a stroke). Cons: Always semi‐rigid (can be uncomfortable and harder to conceal). Brands: Genesis (Coloplast), Spectra (Boston Scientific) How to choose the best one? 1. If you want the most natural look and function: • Choose a three‐piece inflatable implant (AMS 700 or Titan) 2. If you have limited hand dexterity or want a simpler device: Go for a malleable implant 3. If cost is a major factor: Malleable implants are cheaper, but some insurance covers three‐piece options. 4. If you want the longest lifespan and lowest mechanical failure risk: Coloplast Titan has a slightly thicker design, which may be more durable. 5. If you have had prior surgeries or radiation therapy: Discuss with your urologist, as scar tissue may affect which implant works best.	*Clarity* *Medical correctness* *Relevance* *Risk of misinformation*
2. How does the three‐component penile prosthesis work?	Inflating the Implant (Achieving an Erection) The user presses the pump (located in the scrotum). This moves fluid from the reservoir into the cylinders inside the penis, making it hard and rigid like a natural erection. The penis remains erect until the fluid is released. Deflating the implant (returning to a flaccid state) The user presses the release valve (on the pump in the scrotum). The fluid returns from the cylinders back to the reservoir. The penis becomes soft and flaccid again.	Clarity Medical correctness Relevance Risk of misinformation
3. What is the recovery time after penile prosthesis implantation?	The recovery process after penile implant surgery varies based on the type of implant, your overall health, and the surgeon's technique. Generally, here is what to expect: 1. Immediate post‐surgery (0–2 weeks) Hospital stay: Most patients go home the same day or after an overnight stay. Pain and swelling: Moderate pain and swelling for the first 3–7 days, managed with pain medications and ice packs. Catheter: If used, it is usually removed within 24 h. Movement: Limited activity; avoid lifting, bending, or straining. Antibiotics: Prescribed to prevent infection. 2. Early recovery (2–6 weeks) Swelling and bruising decrease significantly. Stitches dissolve within 2–3 weeks. Light activity is allowed, but no heavy lifting or strenuous exercise. No sexual activity or inflation of the implant yet. 3. Full recovery (6–8 weeks) First inflation (for inflatable implants) is typically done by the doctor at 4–6 weeks to teach you how to use it. Sexual activity can usually resume at 6–8 weeks, once healing is complete. Most men return to work (if not physically demanding) within 2–3 weeks; strenuous jobs may require 4–6 weeks. 4. Long‐term outlook Most men are fully comfortable using the device by 3 months. Implants typically last 10–15 years or more, depending on the model and usage.	Clarity Medical correctness Relevance Risk of misinformation
4. What are the recommendations before and after penile prosthesis implantation surgery?	Before surgery: Preparation ✅ Choose an experienced surgeon Pick a high‐volume urologist specializing in penile implants to reduce complications. ✅ Medical clearance Your doctor may require blood tests, EKG, and urine tests to ensure you are fit for surgery. If you have diabetes, ensure good blood sugar control to reduce infection risks. ✅ Stop certain medications Blood thinners (Aspirin, Warfarin, Clopidogrel) may need to be stopped 1 week before surgery (check with your doctor). NSAIDs (Ibuprofen, Naproxen) should also be avoided. ✅ Stop smoking and alcohol Smoking can delay healing and increase infection risk. Avoid alcohol at least 24–48 h before surgery. ✅ Shaving and hygiene Some surgeons may require preoperative shaving of the genital area. Shower with an antibacterial soap the night before and morning of surgery. ✅ Fasting No food or drink 8–12 h before surgery, depending on anesthesia instructions.	Clarity Medical correctness Relevance Risk of misinformation
5. After how long can sexual intercourse be resumed after penile prosthesis implantation?	You can typically **resume sexual activity 6–8 weeks** after penile prosthesis implantation. However, the exact timing depends on **healing progress, implant type, and surgeon's recommendations**. **Why wait 6–8 weeks?** ✅ **Allow tissues to heal** – The body needs time to recover from surgery and avoid complications like infection or implant shifting. ✅ **Reduce pain and swelling** – The surgical area may still be sensitive for the first few weeks. ✅ **Ensure implant function** – At around **4–6 weeks**, your doctor will guide you on **how to inflate and deflate** (for inflatable models) to ensure proper function before use. **Signs you are ready for sex** No pain or swelling in the surgical area. You can **comfortably inflate and deflate** (for inflatable implants). Your doctor gives you the **green light** at a follow‐up visit.	*Clarity* *Medical correctness* *Relevance* *Risk of misinformation*
6. Is it necessary to use erection pills after penile prosthesis implantation?	**No, erection pills (like Viagra, Cialis, or Levitra) are not needed after a penile prosthesis implantation**. **Why not?** 💡 **The implant replaces the natural erection mechanism**. A penile prosthesis (whether inflatable or malleable) **mechanically creates an erection** without requiring blood flow improvement, which is what erection pills do. Pills like Viagra work by **enhancing blood flow**, but implants work independently of blood circulation. 💡 **The prosthesis provides full rigidity on demand**. With an **inflatable implant**, you control when to inflate and deflate. With a **malleable implant**, you simply position the penis as needed. Since you do not need extra blood flow stimulation, **pills will not add any benefit**. **Are there any exceptions?** 🚨 **Only if you still have partial erectile function (rare cases)**. If some natural erections are possible, but you have an implant for **partial assistance**, a doctor might consider combining treatments. This is uncommon because most men getting a penile implant **no longer respond to pills** due to severe ED.	*Clarity* *Medical correctness* *Relevance* *Risk of misinformation*
7. Can you achieve orgasm with a penile prosthesis?	**Yes, you can still achieve orgasm and experience pleasure with a penile prosthesis!** However, this depends on **nerve function, sensation, and overall sexual health** rather than the implant itself. **How does orgasm work after implant surgery?** 💡 **The penile implant only replaces the erection mechanism**, not sensation or orgasm ability. 💡 If you could orgasm before surgery, you **should still be able to orgasm** after the implant. **Factors that affect orgasm after implant surgery** 1⃣ **Nerve function** If your **nerves are intact** (e.g., no major nerve damage from diabetes, prostate surgery, or spinal cord injury), orgasm remains possible. 2⃣ **Sensation** The implant does not affect **penile skin sensation** or the ability to feel pleasure. 3⃣ **Prostate and pelvic surgery history** If you had **radical prostatectomy (prostate removal), pelvic radiation, or nerve damage**, orgasm may feel different but is often still possible. 4⃣ **Psychological factors** Anxiety, depression, or self‐consciousness about the implant may impact orgasm initially. **Will ejaculation still happen?** 💡 **Depends on your medical history**: If your **prostate and seminal vesicles are intact**, you should still ejaculate. If you had a **prostatectomy (prostate removal), you will not ejaculate** but can still orgasm (“dry orgasm”). **Tips to enhance orgasm with a penile implant** ✅ **Foreplay and stimulation** – Mental arousal and physical touch still play a big role. ✅ **Lubrication** – Enhances comfort and pleasure. ✅ **Experiment with different positions** – Some may feel better with the implant. ✅ **Pelvic floor exercises (Kegels)** – Can improve orgasm intensity. ✅ **Patience and communication** – It may take a few attempts to adjust.	*Clarity* *Medical correctness* *Relevance* *Risk of misinformation*
8. Can you resume physical activity after penile prosthesis implantation?	Yes, you can resume physical activity after penile prosthesis implantation, but you need to **follow a gradual recovery plan** to avoid complications. **General timeline for resuming activities** ✅ **First week (0–7 days): Rest and recovery** **No strenuous activities**. Avoid lifting, bending, or excessive movement. **Walking is okay**, but keep it minimal. **Ice packs and scrotal support** to reduce swelling. ✅ **Weeks 2–4: Light activities only** Gentle walking is encouraged. Avoid **cycling, running, or heavy lifting** (puts pressure on the groin area). No swimming, hot tubs, or baths (infection risk). ✅ **Weeks 4–6: Moderate activity** Light cardio like **brisk walking or treadmill** is usually okay. **No core workouts or heavy squats/deadlifts yet**. Still avoid high‐impact activities. ✅ **After 6–8 weeks: Full return to exercise and sports** Most men can return to **gym workouts, swimming, cycling, and sports** after clearance from their doctor. **Sexual activity** is typically allowed after 6–8 weeks. If you do **high‐impact sports (football, martial arts, etc.)**, consider extra protection for the implant area.	*Clarity* *Medical correctness* *Relevance* *Risk of misinformation*
9. How long does a penile prosthesis last? Does a hydraulic penile prosthesis require replacement?	**How long does a penile prosthesis last?** A **penile prosthesis typically lasts 10–15 years**, but it can vary based on: ✅ **Implant quality and brand** (Coloplast Titan and AMS 700 are durable). ✅ **How often it is used** – Frequent inflation/deflation may slightly affect lifespan. ✅ **Surgical technique** – A skilled surgeon ensures proper placement, reducing wear and tear. ✅ **Patient health** – Diabetes, infections, or scar tissue formation can affect longevity. **Does a hydraulic (inflatable) penile prosthesis require replacement?** 🔹 **Yes, but only if there is a problem**. A hydraulic (inflatable) penile implant does **not need routine replacement**, but it may need replacement if: 🚨 **Mechanical failure occurs** – The pump, reservoir, or cylinders can wear out over time. 🚨 **Infection develops** – Although rare (< 3% risk), infection requires removal and replacement. 🚨 **Leaks or malfunctions happen** – Fluid leaks can make inflation difficult or impossible. 🚨 **Scar tissue or pain affects function** – Rare cases may require revision surgery. **Signs that your implant may need replacement** ⚠ Difficulty inflating or deflating ⚠ Loss of rigidity or deflation too quickly ⚠ Pain or discomfort in the implant area ⚠ Visible changes (shifting, uneven cylinders) If the implant is working well, **there is no need for replacement**. Many men keep the same implant for **15+ years** without issues.	*Clarity* *Medical correctness* *Relevance* *Risk of misinformation*
10. After penile prosthesis implantation surgery, will the penis be normal?	**Yes, the penis will look and function normally, but there may be some differences**. Most men report high satisfaction after getting a penile prosthesis, but it is important to understand what to expect. **Appearance: Will it look normal?** ✅ **Natural appearance** – The implant is **completely internal**, so there are **no visible external changes** (except for a small surgical scar). ✅ **Flaccid and erect states** – With a **three‐piece inflatable implant**, the penis appears natural when both flaccid and erect. ❌ **Possible shortening** – Some men experience a slight **loss of length (1–2 cm)** due to fibrosis or tissue changes from prior ED. Regular inflation exercises can help. ❌ **Shaft rigidity only** – The **glans (head) does not become firmer**, since the implant only affects the shaft. Some men use medications like **Trimix or Viagra** for added glans firmness. **Sensation and pleasure: Will it feel normal?** ✅ **Normal skin sensation** – The implant does **not affect nerves**, so you will still feel touch and pleasure. ✅ **Orgasm and ejaculation remain possible** – If you had these functions before surgery, they will remain unchanged. ❌ **No natural blood flow increase** – Since erections are mechanical, there is no natural engorgement, but this does not affect pleasure. **3. Function: Will it work normally?** ✅ **Reliable and on‐demand erections** – No more waiting for pills or worrying about performance. ✅ **Penetration is easy** – The implant provides **good rigidity**, making intercourse possible anytime. ✅ **Durability** – Implants last **10–15+ years** with proper use.	*Clarity* *Medical correctness* *Relevance* *Risk of misinformation*
11. Can the sensitivity of the penis change after the prosthesis is placed?	Yes, **sensitivity can change** after penile prosthesis implantation, but the extent of change varies from person to person. Here is how: **1. What can affect sensitivity?** **Nerve damage**: If you had previous surgeries (like **prostate surgery** or **pelvic radiation**) that affected the nerves, this might reduce sensitivity. However, the implant itself does not typically damage nerves since it is placed in the **corpora cavernosa** (the erectile chambers). **Penile scar tissue (fibrosis)**: Some men develop **scar tissue** around the implant area, which could lead to changes in sensation. Regular use of the implant and following your surgeon's advice on **inflating and deflating** the device can help reduce this risk. **Implant type**: With a **three‐piece inflatable implant**, the penis may not have the same **natural engorgement** as an erection produced by blood flow, and the **glans (head of the penis)** does not get firmer like in a natural erection. This may affect the **overall sensitivity** during sexual activity. **What to expect in terms of sensitivity?** ✅ **Normal sensitivity for most men**: For most men, **skin sensitivity** (touch, friction, and temperature) remains largely unchanged after surgery. The implant only affects the **erectile function**, not the **sensory nerves** in the skin. ✅ **Ejaculation and orgasm sensitivity**: If you were able to achieve **orgasm** before surgery, you should still be able to do so after surgery. However, if you have had a **prostatectomy** or **nerve damage**, your ability to ejaculate might be affected, but you can still achieve a dry orgasm. **3. How to maintain sensitivity after surgery?** **Avoid scar tissue formation**: **Regular inflation** (for inflatable implants) helps keep the tissues in the penis more pliable and can reduce the development of scar tissue around the implant, which could affect sensitivity. **Communicate with your doctor**: If you notice a significant loss of sensation, consult with your doctor. They may suggest ways to manage the issue, such as **nerve regeneration treatments** or **medications** for improved circulation to the area. **Psychological factors**: Sometimes, **anxiety** or **stress** related to the surgery or implant can affect how sensitive you feel emotionally or physically. Over time, as you adjust to the implant, this can improve.	*Clarity* *Medical correctness* *Relevance* *Risk of misinformation*
12. What kind of erection will I be able to have after the placement of a penile prosthesis?	After the placement of a **penile prosthesis**, you will be able to achieve **an erection on demand**, but the **type of erection** and its feel will be different from a natural, blood‐flow‐induced erection. Here is what to expect: **1. Type of erection with a penile prosthesis** **Mechanical erection**: With a penile prosthesis, you will achieve an erection through **mechanical means**, either through an inflatable pump (for a **three‐piece inflatable prosthesis**) or by manually positioning a malleable rod (for a **two‐piece or malleable prosthesis**). These erections are **not caused by natural blood flow** but by the inflation or rigidity of the implant. **Inflatable implant erection**: **Flaccid state**: The penis will be soft when deflated, like a natural flaccid state. **Erect state**: When inflated, the penis will feel **firm** and **rigid**, similar to a natural erection in terms of **erection quality**, but it may feel slightly firmer than a blood‐induced erection. **Malleable implant erection**: **Always rigid**: The penis remains **rigid** and can be manually bent into an erect position. It will not “fluctuate” as a natural erection would, but you will have a constant firmness ready for penetration. **2. How will the erection feel?** **Firmness**: The implant provides **constant rigidity**, so you will be able to engage in intercourse without worrying about loss of erection. The firmness will be reliable, but it might feel a little different than a natural erection. For inflatable implants, you have control over the firmness by adjusting the inflation, while malleable implants remain firm but allow for manual adjustments. **Size**: **Inflatable implants** may give you a natural feeling **in terms of size and appearance**, as you can control how much you inflate the device to your desired size. **Malleable implants** offer a consistent size and shape but might be **slightly smaller** compared with natural erections. **No blood flow**: Since the prosthesis works mechanically, there is no **natural blood flow** into the penis, so you might not experience the same **increased sensation in the head of the penis** that occurs during a natural erection. Some men report a **slightly reduced sensitivity**, but this varies. **Will you experience natural erection feelings?** **Not exactly**: While the **rigidity** of the implant mimics the physical aspect of a natural erection, you **will not experience the same sensation** as blood‐filled erections. The **glans (head of the penis)** may not feel as **firm** or **sensitive** as it would with a natural erection. However, **penile sensitivity** (touch, stimulation, etc.) typically remains unchanged because the nerves and skin are unaffected by the implant. **4. Orgasm and ejaculation** **Orgasm**: You can still achieve orgasm, just like before the implant. However, men who have had prostate surgery (prostatectomy) may not ejaculate (dry orgasm), but they can still experience **pleasure and orgasmic sensations**. **Ejaculation**: Men with intact **prostate and seminal vesicles** will still be able to ejaculate. If you have had a **prostatectomy** (removal of the prostate), you will not ejaculate but can still experience orgasm.	*Clarity* *Medical correctness* *Relevance* *Risk of misinformation*
13. How long does sexual intercourse last with a penile prosthesis?	The **duration of sexual intercourse** with a penile prosthesis can vary depending on several factors, but in general, a penile prosthesis provides **consistent and reliable erections**, allowing for a normal or satisfying sexual experience. . **Erection quality**: • With an **inflatable prosthesis**, the penis becomes fully rigid when inflated, allowing for sustained intercourse. You can control the firmness of the erection, so the duration depends on your comfort and stamina. • A **malleable prosthesis** stays firm, but you manually adjust the position as needed. Again, duration depends on individual factors. 2. **Personal stamina**: • The implant allows for a **consistent erection**, so the actual length of intercourse is determined more by your **stamina, arousal, and overall sexual health** than the implant itself. 3. **Partner interaction**: • Like with natural erections, the length of time will vary depending on **arousal, foreplay, and overall sexual satisfaction**. Communication with your partner also plays a key role in ensuring a satisfying experience. 4. **Implant type**: • **Inflatable implants** are designed to mimic a natural erection and can remain firm for as long as needed, although some men may deflate the implant after intercourse for comfort. • **Malleable implants** are always firm, so the duration of intercourse depends on comfort and stamina. **Typical duration of intercourse with a penile prosthesis**: • **Intercourse duration** may last anywhere from **10 min to 30 min**, depending on factors like arousal, desire, stamina, and how comfortable you are with the implant. Some men may enjoy longer sessions without any loss of rigidity, thanks to the reliable support of the prosthesis. • Since the prosthesis offers consistent rigidity, men report fewer concerns about **erection loss** during intercourse, which can contribute to a more **relaxed and enjoyable experience**.	*Clarity* *Medical correctness* *Relevance* *Risk of misinformation*
14. What are the potential complications of penile prosthesis surgery?	While penile prosthesis surgery is generally safe and effective, like any surgical procedure, it comes with potential risks and complications. Understanding these can help you manage expectations and address any concerns with your doctor. Here are the main possible complications: **1. Infection (rare)** • **Risk**: Infections are a rare but serious complication, typically occurring within the first few weeks after surgery. • **Signs**: Redness, swelling, fever, pain, or discharge at the surgical site. • **Management**: If an infection occurs, the prosthesis may need to be removed, and antibiotics are typically used. In some cases, the implant can be replaced after the infection is cleared. **2. Mechanical failure (rare)** • **Risk**: The prosthesis components (pump, reservoir, or cylinders) may malfunction or break over time, especially in inflatable implants. • **Signs**: Difficulty inflating or deflating, loss of rigidity, or an inability to fully deflate the implant. • **Management**: If mechanical failure occurs, the prosthesis may need to be repaired or replaced. Mechanical issues tend to occur **years after surgery** (typically 10–15 years), so routine maintenance or use can extend the implant's lifespan. **3. Erosion (rare)** • **Risk**: Erosion occurs when the implant rubs against surrounding tissue, potentially causing the implant to push through the skin or damage nearby structures. • **Signs**: Pain, swelling, or leakage of fluid. • **Management**: Erosion may require surgical removal of the implant and the use of a new one, once the area has healed. **4. Hematoma (blood collection)** • **Risk**: A collection of blood may form around the surgical site. • **Signs**: Swelling, bruising, or pain around the implant area. • **Management**: A hematoma typically resolves on its own, but in severe cases, drainage may be required. It is important to follow post‐surgery care to reduce this risk. **5. Scarring (fibrosis)** • **Risk**: Scar tissue (fibrosis) can form around the implant, which may cause the penis to feel firmer or reduce the range of motion. • **Signs**: Hardness or discomfort around the implant area. • **Management**: Scar tissue is a common side effect, but it can often be managed with regular use of the implant and may improve over time. In rare cases, further surgical intervention may be needed. **6. Pain or discomfort** • **Risk**: Some discomfort or pain in the groin or penis area may occur during the early recovery period. • **Signs**: Sensitivity, pain when inflating the implant, or soreness in the surgical area. • **Management**: Pain can typically be managed with medication, but it usually resolves within a few weeks as healing progresses. If pain persists or worsens, a follow‐up with your surgeon is recommended. **7. Penile length changes (shortening)** • **Risk**: Some men report a slight reduction in penile length after implantation due to tissue scarring or changes in the way the implant fits. • **Signs**: A slightly shorter penis when flaccid or erect. • **Management**: While penile length loss is usually minimal, some men can use an inflatable implant with adjustable cylinders to optimize length. **8. Difficulty inflating/deflating (for inflatable implants)** • **Risk**: Inflatable implants rely on a pump system to inflate and deflate. If the pump malfunctions, you might have difficulty achieving an erection or deflating the implant.	*Clarity* *Medical correctness* *Relevance* *Risk of misinformation*
	• **Signs**: Difficulty using the pump or the inability to return the implant to its flaccid state. • **Management**: In most cases, mechanical issues can be resolved through revision surgery or adjusting the pump system. **9. Psychological impact** • **Risk**: Some men experience **anxiety, depression, or dissatisfaction** due to the change in their sexual function or appearance after the surgery. • **Signs**: Emotional distress, frustration with implant use, or self‐esteem concerns. • **Management**: Psychological support, counseling, or support groups can be helpful. Open communication with your partner and healthcare provider can also improve satisfaction. **Prevention and minimizing complications**: 1. **Follow post‐surgery instructions**: Carefully follow your surgeon's advice on **activity restrictions, wound care, and hygiene** to reduce the risk of infection and complications. 2. **Regular checkups**: Regular follow‐ups with your doctor will help monitor the implant's function and address any issues early. 3. **Gradual return to physical activity**: After surgery, gradually return to physical activities as recommended to avoid unnecessary strain on the surgical site.	
15. Is penile prosthesis placement surgery reversible?	No, **penile prosthesis implantation surgery is generally not reversible**. Once the prosthesis is implanted, the procedure is considered **permanent**, and the prosthesis cannot be removed and replaced with a natural erection function. Here is why: **Why is it not reversible?** 1. **Permanent implant placement**: During the surgery, the prosthesis (whether inflatable or malleable) is surgically **placed inside the erectile tissue** of the penis. This requires **tissue manipulation and sometimes removal of damaged erectile tissue** (such as in cases of erectile dysfunction that did not respond to other treatments). The **implant stays inside** permanently unless it fails, becomes infected, or requires removal due to complications. 2. **Loss of natural erection function**: The prosthesis provides a mechanical solution to erectile dysfunction (ED). Once it is implanted, the **natural blood flow mechanism** of the penis (which causes natural erections) is bypassed. This means the **ability to get an erection without the prosthesis is generally lost**. In rare cases, the implant may be removed due to infection or mechanical failure, but the natural erectile function may not be restored after the removal. **Tissue changes** caused by the implant (such as fibrosis or scarring) can make it difficult for the penis to function normally without the prosthesis. 3. **Potential for prosthesis removal**: If there is a **complication** like infection, mechanical failure, or erosion of the prosthesis, it may need to be **removed**. However, this does not **restore natural erectile function**. **Reconstruction surgery** or other options like **medications or injections** might be needed for sexual function after removal, but they will not be able to fully replicate the natural erectile process. **What happens if the prosthesis needs to be removed?** **After removal**, the **penis may be left flaccid**, and achieving natural erections may not be possible without additional treatments. **Alternative treatments** such as **penile injections, vacuum erection devices (VEDs)**, or **other prosthetic devices** may be recommended, but **natural erections are unlikely to return**. **Penile reconstruction** surgeries or **other options** (like vascular surgery or medications) might help with some cases, but these solutions depend on individual circumstances.	*Clarity* *Medical correctness* *Relevance* *Risk of misinformation*
